# Clinical analysis of 11 cases of nocardiosis

**DOI:** 10.1515/med-2020-0196

**Published:** 2021-04-08

**Authors:** Yiqing Li, Ting Tang, Jie Xiao, Jieyu Wang, Boqi Li, Liping Ma, Shuangfeng Xie, Danian Nie

**Affiliations:** Department of Hematology, Guangdong Provincial Key Laboratory of Malignant Tumor Epigenetic and Gene Regulation, Sun Yat-Sen University, Sun Yat-Sen Memorial Hospital, Guangzhou, Guangdong 510120, People’s Republic of China

**Keywords:** *Nocardia* infections, immunosuppressive agents, diagnosis, therapeutics

## Abstract

Nocardiosis is a rare, life-threatening, opportunistic, and suppurative infection. Its clinical manifestation lacks specificity, which makes early diagnosis difficult. A retrospective analysis of the clinical records of 11 patients with nocardiosis admitted to our hospital from January 2013 to November 2018 was conducted. All patients had at least one underlying disorder, such as an autoimmune disease (6/11), a blood malignancy (2/11), avascular necrosis of the femoral head (1/11), bronchiectasis (1/11), or pneumonia (1/11). The first-line treatment was trimethoprim–sulfamethoxazole (TMP–SMX); one or two additional antibiotics were given according to the drug-sensitive test. The median time from onset to treatment was 3 weeks (ranging from 1 to 9 weeks). The median duration of treatment after diagnosis was 20.5 weeks (ranging from 7 to 47 weeks). Eight patients were discharged and survived, and three patients died. This indicates that early use of TMP–SMX combined with sensitive antibiotics could improve the condition of patients and improve the cure rate (8/11). Clinically, it is necessary to consider the possibility of nocardiosis in patients with long-term use of immunosuppressants and poor response to treatment of common bacterial infections. Early diagnosis, timely treatment, and combination drug therapy are keys to improving the outcomes of patients with nocardiosis.

## 1 Introduction

The pathogen of nocardiosis is *Nocardia*, which is a slow-growing gram-positive aerobic bacterium with acid-fast staining properties [1]. *Nocardia* is an opportunistic pathogen that can cause local or systemic suppurative infections in humans and animals, some of which are life-threatening [2]. The clinical features of nocardiosis always lack specificity, making early diagnosis difficult. The gold standard for diagnosis of *Nocardia* infection is based on the isolation and identification of *Nocardia* via 16S ribosomal ribonucleic acid (rRNA) sequencing from humoral secretions or tissues, such as blood, sputum, pus, pleural effusion, cerebrospinal fluid, and pulmonary puncture samples [3,4,5]. A full 2–3 weeks may lapse between specimen collection and detection of *Nocardia* [1,6]. *Nocardia* spp. can be classified into different species with the comprehensive application of biochemical techniques, including 16S rRNA PCR-based assays and multi-site sequencing analysis [3,7,8]. Because of the relatively long duration and strict requirements for *Nocardia* detection, gene sequencing is not always available. In our report, the strains of *Nocardia* in six patients could not be identified because of the limitations of the experimental conditions.

Positive results for nocardiosis are frequently followed by immediate appropriate treatment and prolonged maintenance therapy. In terms of treatment for *Nocardia* infection, the optimal therapeutic agent, route of administration, and duration of treatment have not been well established. Most treatments are based on the results of basic research, animal models, and recommendations from experts [7,9]. Trimethoprim–sulfamethoxazole (TMP–SMX) and linezolid have strong inhibitory effects on *Nocardia in vitro* [3]. TMP–SMX can be used as an initial empirical treatment [10,11]. Different strains of *Nocardia* have different levels of antimicrobial resistance, and some of them may be resistant to sulfonamides [12,13]. It is particularly important to identify *Nocardia* and perform drug-sensitive tests. In 2011, the Clinical and Laboratory Standards Institute M24-A2 guidelines published an approved broth-microdilution method for susceptibility testing of aerobic actinomycetes [14]. The first-line medications include TMP–SMX, oxazolidinones (linezolid), aminoglycosides (amikacin and tobramycin), carbapenems (imipenem), ß-lactams (ceftriaxone and amoxicillin-clavulanic acid), macrolides (clarithromycin), quinolones (moxifloxacin, ciprofloxacin, and levofloxacin), and tetracyclines (minocycline). The second-line medications include cephalosporins (cefepime and cefotaxime) and tetracyclines (doxycycline). For patients with disseminated diseases, central nervous system (CNS) involvement, and/or severe *Nocardia* infection, a three-drug regimen including TMP–SMX, amikacin, and imipenem or ceftriaxone is recommended [8,15]. Although the duration of treatment for nocardial infections is unclear, 6 weeks of treatment for topical nocardiosis and 6 months to 1 year of treatment for systemic nocardiosis are recommended. The duration of treatment depends on the response to the therapy and the immune function of the patient [8,15].

In this study, we retrospectively analyzed the clinical records of patients with nocardiosis who were admitted to our hospital in the last 5 years (January 2013 to November 2018). We also reviewed the relevant literature to provide references for early diagnosis and treatment of nocardiosis.

## 2 Methods

In this retrospective study, 11 patients with nocardiosis from our hospital who were diagnosed with conventional phenotypic and biochemical species identification were included. Over the 5-year period from January 2013 to November 2018, demographic data (such as age, sex, underlying diseases, and risk factors), clinical manifestations, radiological investigation, pathology features, treatment, and patient outcomes were reviewed. Mixed infection was considered if evidence of infection of microorganisms other than *Nocardia* was found 7 days before or 7 days after the date of the nocardiosis diagnosis. The research related to human use has been approved by the Medical Ethics Committee of Sun Yat-sen Memorial Hospital.

### 2.1 Statistical analysis

Statistical analyses were performed using Statistical Product and Service Solutions (SPSS) Software, version 25, to calculate the median.

## 3 Results

### 3.1 Demographic data and underlying diseases

Eleven patients (seven males and four females) were diagnosed with nocardiosis (Table 1). The median age was 42 years (12–78 years). All patients had at least one underlying disease, such as an autoimmune disease (6/11), blood malignancy (2/11), avascular necrosis of the femoral head (1/11), bronchiectasis (1/11), and pneumonia (1/11). Immunosuppressive or cytotoxic agents were used in eight patients. The median hospitalization time was 23 days (6–58 days). Six patients received invasive procedures. Mixed infection was present in three patients: *Acinetobacter baumannii*, *Candida albicans*, and *Proteusbacillus vulgaris* infection.

**Table 1:** Demographic and underlying disease for the patients with nocardiosis

No.	Age (years)	Gender	Diagnosis	Underlying diseases	Immunosuppressant or chemotherapy	Hospitalization time (days)	Invasive procedures
1	26	Male	Disseminated nocardiosis (*Nocardia farcinica*) (lung, head, blood)	Undifferentiated connective tissue disease, cerebral vasculitis	Methylprednisolone, azathioprine	28	None
2	53	Female	Pulmonary nocardiosis (*Nocardia otitidiscaviarum*)	Bronchiectasis	None	7	None
3	42	Male	Pulmonary nocardiosis	Nephrotic syndrome, diabetes mellitus	Methylprednisolone, cyclosporine	58	Trachea cannula, pleural drainage, CVC, bronchofiberscope
4	57	Male	Left hip joint nocardiosis (*Nocardia brasiliensis*)	Avascular necrosis of the femoral head	None	52	Arthroscopy, articular cavity cleaning
5	23	Male	Disseminated nocardiosis (skin, blood)	Systemic lupus erythematosus, lupus nephritis, generalized psoriasis	Methylprednisolone, hydroxychloroquine	15	None
6	78	Male	Disseminated nocardiosis (*Nocardia asteroids*) (lung, blood)	Adult onset Still’s disease	Methylprednisolone, cyclosporine	6	None
7	22	Female	Skin nocardiosis	Systemic lupus erythematosus, lupus nephritis, lupus gastrointestinal damage	Prednisone, hydroxychloroquine, methotrexate	36	Incision and drainage for abscesses
8	58	Female	Disseminated nocardiosis (lung, skin, abdominal cavity)	Systemic lupus erythematosus, lupus nephritis, lupus blood system damage, lupus cardiac system damage; secondary Sjogren’s syndrome	Methylprednisolone, cyclosporine, hydroxychloroquine	15	None
9	56	Male	Pulmonary nocardiosis	Pneumonia	None	22	Bronchoscope submucosal biopsy, lung puncture biopsy
10	12	Female	Disseminated nocardiosis (skin, lung)	Acute myelogenous leukemia (M1)	IA, MA	34	Skin biopsy, nodule biopsy, PICC
11	34	Male	Disseminated nocardiosis (*Nocardia reynolds*) (skin, soft tissue, lung)	T lymphocytic lymphoma, post-allogeneic HSCT, chronic graft versus host disease	Methylprednisolone, cyclosporine	23	Abscess incision, abscess debridement exploration

Abbreviations: IA: idarubicin, cytarabine. MA: mitoxantrone, cytarabine. HSCT: hematopoietic stem cell transplantation. CVC: central venous catheter. PICC: peripherally inserted central catheter.

### 3.2 Clinical features and auxiliary examination

**Figure 1:**
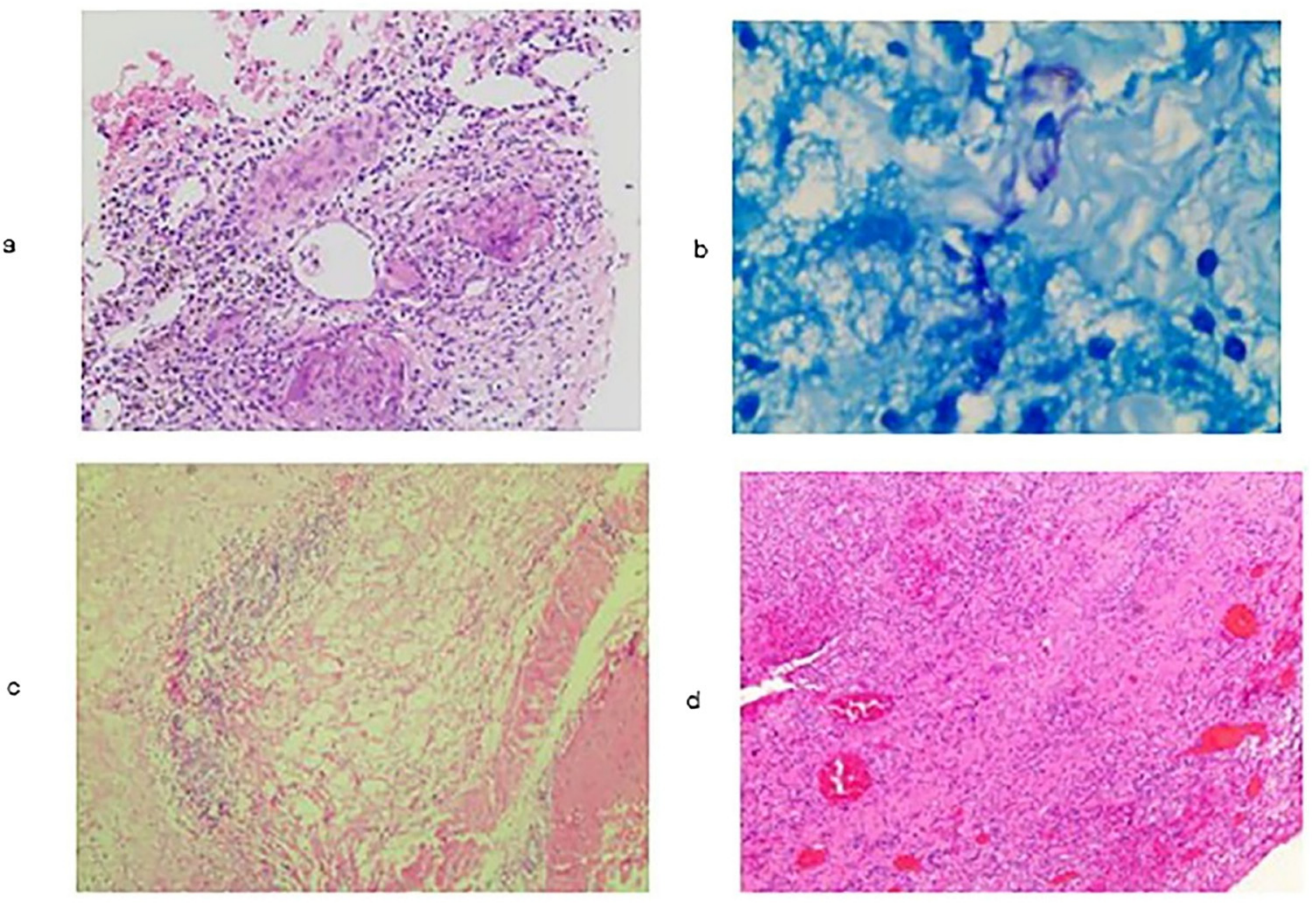
Pathological features of nocardiosis patients. (a) The histopathology of the right lung mass of patient No. 9 showed chronic granulomatous inflammation and hyperplasia of fibrous tissue and lymphoid tissue with necrosis (HE staining, ×200 magnification). (b) Acid-fast staining of the left calf gastrocnemius muscle of the patient No. 10. The result was suspected to be positive (×400 magnification). (c) The histopathology of the gastrocnemius muscle in the left leg of patient No. 10 suggested some neutrophil infiltration and a small number of mild atypical cells, consistent with suppurative inflammation (HE staining, ×100 magnification). (d) The histopathology of the abscess in the left upper limb of patient No. 11 showed chronic purulent inflammation, hyperplasia of fibrous and granulation tissue, and more purulent exudate (HE staining, ×100 magnification).

The clinical manifestations of nocardiosis were variable, nonspecific, and heterogeneous (Table 2). The most common symptoms were fever (10/11), cough (7/11), expectoration (7/11), and pain (7/11) including joint pain (2/11), chest pain (2/11), headache (1/11), back pain (1/11), and abdominal pain (1/11). Chest tightness, shortness of breath, weight loss, and fatigue were also frequently noted. In patients with pulmonary nocardiosis, mass shadows (4/11), pleural effusion (3/11), multiple nodules (2/11), cavities (2/11), and bronchiectasis (1/11) were shown by chest radiography. Routine blood examinations revealed seven cases of leucocytosis. The pathological features were neutrophil infiltration, suppurative or granulomatous inflammation, and a suspiciously positive reaction to acid-fast staining (Figure 1).

**Table 2:** Clinical, laboratory, and radiological features of the patients

No.	Diagnosis	Clinical manifestations	Blood routine			Radiographic findings
			WBC (×10^9^/L)	Neu (×10^9^/L)	PCT (ng/mL)	
1	Disseminated nocardiosis (*Nocardia farcinica*) (lung, head, blood)	Fever (39.6°C), cough, expectoration, headache	10.9	10.46	0.18	Chest CT showed multiple patchy, mass dense shadows and cavities
2	Pulmonary nocardiosis (*Nocardia otitidiscaviarum*)	Fever (39.0°C), cough, expectoration, blood-stained sputum, chest tightness	4.36	2.66	None	Chest CT showed multiple bronchiectasis with infection
3	Pulmonary nocardiosis	Fever (39.5°C), cough, expectoration, chest tightness, chest pain, shortness of breath	10.25	8.8	5.8	Chest CT showed multiple nodules, cavities, and pleural effusion
4	Left hip joint nocardiosis (*Nocardia brasiliensis*)	Left hip pain, weight loss	7.37	5.51	0.42	X-ray of hip joint showed ischemic necrosis combined with osteoarthritis on bilateral femoral head
5	Disseminated nocardiosis (skin, blood)	Fever (39.6°C), skin erythema, desquamation and pruritus, back pain	11.46	9.9	0.14	Lumbar X-ray and chest X-ray showed no abnormalities
6	Disseminated nocardiosis (*Nocardia asteroids*) (lung, blood)	Fever (39.4°C), chills, cough, expectoration, limbs weakness	10.38	10.06	None	Chest X-ray showed multiple cloud-like mass shadows
7	Skin nocardiosis	Fever (38.2°C), abdominal pain, vomiting, fatigue, purulent, and ulcerated on right foot	10.69	10.18	None	Chest X-ray and abdominal ultrasound showed no abnormalities
8	Disseminated nocardiosis (lung, skin, abdominal cavity)	Fever (40.0°C), cough, expectoration, skin abscess, abdominal distension	7.1	6.63	0.25	Chest CT showed double pneumonia and pleural effusion
9	Pulmonary nocardiosis	Fever (39.0°C), cough, expectoration, chest tightness, chest pain, shortness of breath, weight loss	27.75	24.21	1.07	PET–CT showed massive hypermetabolic lesions, multiple strips, and mass shadows around the lesion, pleural effusion
10	Disseminated nocardiosis (skin, lung)	Fever (40.0°C), skin abscess in both lower extremities	4.45	3.08	0.1	Chest CT showed high-density shadow and exudation
11	Disseminated nocardiosis (*Nocardia reynolds*) (skin, soft tissue, lung)	Fever (38.6°C), cough, expectoration, pain on left elbow	13.4	8.13	0.17	Chest CT showed multiple nodules

Abbreviations: WBC: white blood cell. N: neutrophilia cell. PCT: procalcitonin. CT: computed tomography. PET–CT: positron emission tomography-computed tomography.

### 3.3 Diagnosis, treatment, and outcomes

The diagnoses of all patients were confirmed by microbiologic studies. Patient specimens were obtained from blood (4/11), sputum (2/11), pus (2/11), joint fluid (1/11), and biopsy (1/11). Using 16S rRNA gene polymerase chain reaction (PCR) for species identification, 5 of 11 cases were classified into specific genotypes: *N. asteroids*, *N. otitidiscaviarum*, *N. brasiliensis*, *N. farcinica*, and *N. Reynolds*. Two cases were initially misdiagnosed as pulmonary tuberculosis (Table 3).

**Table 3:** Antibiotic regimens and outcomes of the patients

No.	Diagnosis	Diagnostic approach	Misdiagnosis	Time from onset to treatment (weeks)	Treatment time after diagnosis (weeks)	Therapeutic response time (weeks)	Antimicrobials before diagnosis	Antimicrobials after diagnosis	Outcomes
1	Disseminated nocardiosis (*Nocardia farcinica*) (lung, head, blood)	Blood culture	None	2	47	8	Meropenem, Linezolid	TMP–SMX, Meropenem	Improved and survival
2	Pulmonary nocardiosis (*Nocardia otitidiscaviarum*)	Sputum culture	None	1	17	3	Piperacillin Sodium and Sulbactum sodium	TMP–SMX, Piperacillin tazobactam	Improved and survival
3	Pulmonary nocardiosis	Sputum culture	None	3	7	2	Linezolid	TMP–SMX, Meropenem	Improved and survival
4	Left hip joint nocardiosis (*Nocardia brasiliensis*)	Joint fluid culture	None	9	20	1	Cefuroxime sodium, Doxycycline, Vancomycin	TMP–SMX, Rifampicin, Streptomycin	Improved and survival
5	Disseminated nocardiosis (skin, blood)	Blood culture	None	2	23	1	Cefoperazone Sodium and Sulbactam Sodium	TMP–SMX, Levofloxacin	Improved and survival
6	Disseminated nocardiosis (*Nocardia asteroids*) (lung, blood)	Blood culture	Pulmonary tuberculosis	3	Unknown	Unknown	Meropenem, Linezolid	TMP–SMX, Imipenem, Minocycline	Discharged to another hospital and died
7	Skin nocardiosis	Pus culture	None	8	40	4	Cefoperazone Sodium and Sulbactam Sodium, Ornidazale	TMP–SMX, Streptomycin, Moxifloxacin	Improved and survival
8	Disseminated nocardiosis (lung, skin, abdominal cavity)	Blood culture	None	2	Unknown	Unknown	Meropenem	TMP–SMX, Levofloxacin	Discharged to home and died
9	Pulmonary nocardiosis	Tissue biopsy	Pulmonary tuberculosis	9	Unknown	Unknown	Amikacin, Levofloxacin	TMP–SMX Linezolid, Imipenem	Discharged to home and died
10	Disseminated nocardiosis (skin, lung)	Diagnostic therapy	None	2	21	4	Cefoperazone Sodium and Sulbactam Sodium, Vancomycin	TMP–SMX, Linezolid	Improved and survival
11	Disseminated nocardiosis (*Nocardia reynolds*) (skin, soft tissue, lung)	Pus culture	None	3	12	1	Piperacillin Sodium and Sulbactum sodium	TMP–SMX, Linezolid, Levofloxacin	Improved and survival

In all cases, treatment was initiated after diagnosis based on TMP–SMX combined with one or two antibiotics according to the results of drug-sensitive tests. These additional antibiotics were carbapenems (4/11), quinolones (4/11), oxazolidinones (3/11), streptomycin (2/11), tetracycline (1/11), piperacillin ß-lactams (1/11), and rifampicin (1/11). Six patients (6/11) were treated with two antibiotics, and five patients (5/11) were treated with three antibiotics. Most of the patients received antibiotic therapy for a prolonged period.

The median time from onset to treatment was 3 weeks (1–9 weeks), and the median duration of treatment after diagnosis was 20.5 weeks (7–47 weeks). The defined response time to *Nocardia* treatment mainly referred to the time when the clinical symptoms began to abate, including improvement confirmed by chest imaging, a return to normal body temperature, and a reduction in other clinical symptoms. In patients who responded to anti-*Nocardia* treatment, the median response time was 2.5 weeks (1–8 weeks). Regarding outcomes, eight patients were discharged after improvement and survived, and three patients died (Table 3).

## 4 Discussion

*Nocardia* is a genus of prokaryotes, firmicutes, actinomycetes, and gram-positive aerobic bacteria. This genus is widely distributed in the environment (e.g., soil, water, air, grass, and rotting plants), and most of the species are saprophytic non-pathogenic bacteria [4]. Since the French veterinarian Edmund Nocard first discovered them in 1888 [15], more than 100 species of *Nocardia* have been reported [1]. The important pathogens involved in human nocardiosis are *Nocardia asteroids*, *Nocardia brasiliensis*, *Nocardia farcinica*, *Nocardia cyriacigeorgica*, and *Nocardia otitidiscaviarum*. *N. asteroids* is the most common isolated species [7,16].

*Nocardia* is a genus of opportunistic pathogens that mainly affects patients with deficient cellular immunity, such as those with a history of long-term steroid or immunosuppressant use, organ or stem cell transplantation, diabetes, acquired immune deficiency syndrome (AIDS), or chronic lung disease [6,9]. As widely described in the literature, the administration of corticosteroids and/or immunosuppressants is the most common predisposing factor [17,18]. Eight patients in our report received immunosuppressive or cytotoxic agents, which was consistent with previous reports and indicated that nocardiosis is an opportunistic infection usually occurring in patients with immune deficiencies.

*Nocardia* can spread to almost all parts of the body through the blood from the lungs (especially the upper lobes of the lungs) or infected areas of the skin. As inhalation is the main route of transmission for *Nocardia*, the respiratory tract is the most affected organ, followed by the CNS, skin and soft tissue, kidneys, and peritoneum [19,20,21]. The symptoms of CNS infection include headache, meningeal irritations, seizures, and focal neurological dysfunction. The main manifestations of skin and soft tissue infection are local abscesses [3,5,8]. In this study, 8 of 11 patients (72.73%) had pulmonary infections and presented with cough, expectoration, chest pain, and hemoptysis. Six patients showed disseminated nocardiosis involving lung, blood, joint, head, skin, and soft tissue. All six patients had received corticosteroid or immunosuppressant treatment. The radiological abnormalities in pulmonary nocardiosis are diverse, not pathognomonic, and may mimic a multitude of pulmonary diseases. As the clinical symptoms are similar to tuberculosis, pulmonary nocardiosis is easy to misdiagnose as pulmonary tuberculosis when cultures are not available or confirmative. Two patients in our study (No. 6 and 9) were initially misdiagnosed with pulmonary tuberculosis and received anti-tuberculosis treatment before nocardiosis was confirmed. Both patients died as a result of the progression of nocardiosis, suggesting that misdiagnosis and delayed treatment could cause patient deaths.

**Figure 2:**
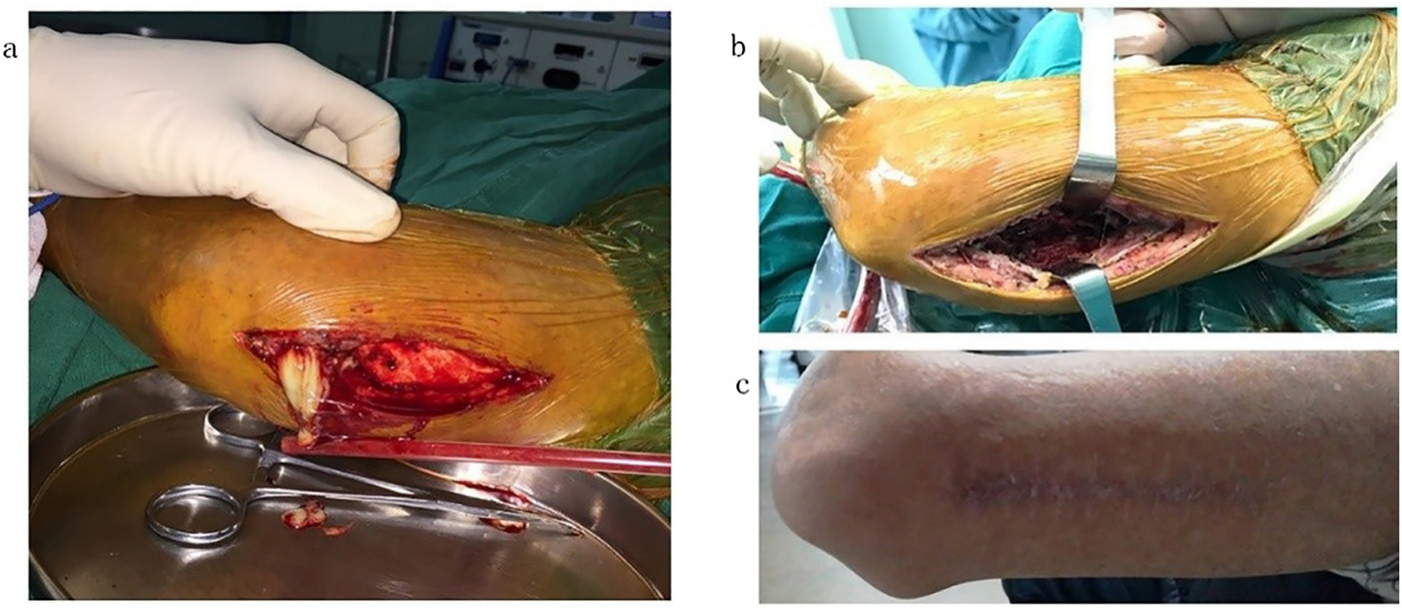
Abscess on the left upper limb of patient No. 11. (a and b) Surgical debridement of the abscess on the left upper limb was performed. (c) The photograph of the left upper limb 12 months after surgery showed that the wound was entirely healed.

In our study, all patients received combination therapy with antibiotics. Eight patients were discharged from the hospital after timely diagnosis and treatment. Patient No. 11 was a recipient of allogeneic hematopoietic stem cell transplant (HSCT) with chronic graft-versus-host-disease (cGVHD) and was receiving corticosteroids and cyclosporins. He was admitted to the hospital with fever, swelling, and pain in the left upper limb and trunk. An incision to drain an abscess on the left upper limb was performed (Figure 2). A bacterial culture of pus suggested nocardiosis (identified and classified as *Nocardia reynolds* several days later). Then, TMP–SMX and levofloxacin were given. After 3 days, linezolid was added according to the results of a drug-sensitive test. The symptoms of the patient significantly regressed with no new lesions occurring 1 week after treatment. The duration of therapy was 12 weeks. No relapse was noted after 12 months. Patient No. 10 was a 12-year-old female with acute myeloid leukemia. She complained of fever and painful swelling erythematous lesions on both lower extremities. Bacterial cultures of pus were repeatedly performed, but the results were negative. A biopsy of the left gastrocnemius muscle was performed, displaying neutrophil infiltration, which was consistent with suppurative inflammation. Acid-fast staining was also suspiciously positive. TMP–SMX plus linezolid was used as a diagnostic treatment. The lesions on the lower extremities healed significantly. The duration of therapy was 21 weeks. She had no evidence of recurrence after an 18-month follow-up. Three patients died. One patient (No. 8), who had systemic lupus erythematosus (SLE), was admitted to the hospital with fever, cough, and expectoration. A blood culture was performed immediately, and a positive result of *Nocardia* was reported 6 days later. She was diagnosed with disseminated *Nocardia*. TMP–SMX and levofloxacin were given initially, but her condition worsened. After 3 days, the drug-sensitive test indicated that the bacterium was sensitive to linezolid and aminoglycoside, but the patient gave up and was discharged from the hospital for economic reasons. Active stage SLE, bloodstream infection, and no use of sensitive antibiotics contributed to the death of the patient. The other two patients did not receive anti-*Nocardia* treatment because they were initially misdiagnosed with pulmonary tuberculosis. Both of these patients died because of delayed treatment of *Nocardia*. Therefore, our study suggests that nocardiosis should be considered in patients with impairment of immunity, particularly in those who do not respond to routine antibiotic therapy. Misdiagnosis and inappropriate management may cause poor outcomes.

In summary, nocardiosis is a relatively rare, opportunistic infection with variable clinical manifestations. Nocardiosis should be considered in patients with infections that rapidly progress or who respond poorly to treatment for common bacterial infections, especially those with a history of long-term steroid and/or immunosuppressant use. In this study, symptoms improved quickly after initiation of therapy based on TMP–SMX combined with carbapenems or other antibiotics according to drug sensitivity in 8 of 11 *Nocardia* infections. Our work suggests that early diagnosis, timely treatment, and combination drug therapy are keys to improving patient outcomes.
